# TL1A Increases Expression of CD25, LFA-1, CD134 and CD154, and Induces IL-22 and GM-CSF Production from Effector CD4 T-Cells

**DOI:** 10.1371/journal.pone.0105627

**Published:** 2014-08-22

**Authors:** Kirsten Reichwald, Tina Z. Jørgensen, Søren Skov

**Affiliations:** Section for Experimental Animal Models, Faculty of Health and Medical Sciences, University of Copenhagen, Copenhagen, Denmark; Institut National de la Santé et de la Recherche Médicale U 872, France

## Abstract

Elevated levels of the cytokine TL1A is associated with several autoimmune diseases e.g. rheumatoid arthritis and inflammatory bowel disease. However, the exact role of TL1A remains elusive. In this study, we investigated the function of TL1A in a pro-inflammatory setting. We show that TL1A together with IL-12, IL-15 and IL-18 increases expression of the co-stimulatory molecules CD154 (CD40 ligand) and CD134 (OX40) on previously activated CD4^+^ T cells. This indicates that TL1A functions as a co-stimulatory molecule, decreasing the activation threshold of T-cells. We have previously shown that TL1A co-stimulation strongly induces IL-6 in human healthy leukocytes. Interestingly, the cytokine-activated effector T-cells did not produce IL-6 in response to TL1A, indicating distinct effects of TL1A on different cell populations. We further show that this co-stimulation increases the expression of CD25 (IL-2Rα) and CD11a (α-chain of LFA-1) on CD4 T-cells, likely governing increased IL-2/IL-15 sensitivity and cell-cell contact. Along with this, TL1A co-stimulation caused a specific induction of IL-22 and GM-CSF from the activated T-cells. These results substantially contribute to the explanation of TL1A's role in inflammation. Our results suggest that TL1A should be considered as a target for immunotherapeutic treatment of rheumatoid arthritis and inflammatory bowel disease.

## Introduction

Cytokine activation or bystander activation has been observed for years, but the mechanisms skewing the regulatory/inflammatory balance have received increased attention during the last decade. Cytokine mediated activation has mainly been described for CD8 or NK/NKT cells, whereas CD4 T-cells have been given less attention [1]–[3]. However, CD4 T-cells are prominent regulators of the immune response which can result in either inflammation or tolerance; this delicate balance is disrupted and tilted towards inflammation in autoimmune diseases. Generally, bystander activation of CD4 T-cells could be the culprit of a range of inflammatory diseases, since the elevated levels of pro-inflammatory cytokines might maintain a feedback loop of co-stimulatory molecules and activating factors, leading to chronic inflammation.

The initial activation of antigen presenting cells causes the production of IL-12, IL-15 and IL-18, cytokines that are often elevated in autoimmunity [4]. These cytokines synergize in inducing IFN-γ production from NK, NKT and T cells, and IL-15 drives growth of NK- and memory CD8-cells [3], [5]. TL1A is a pro-inflammatory cytokine that is found elevated in several diseases such as Rheumatoid Arthritis (RA), Psoriasis and Inflammatory Bowel Disease (IBD) [6]–[8]. It was initially described as a T-cell co-stimulator, and it's potential in inflammation was immediately recognized [9]. In combination with IL-12 and IL-18, TL1A supports IFN-γ production by T cells and NK cells [10], and induces proliferation of human NK, NKT and other T cells in vitro [11]–[14]. We have recently shown that TL1A together with IL-12, IL-15 and IL-18 induces IL-6 and TNF-α production in leukocytes purified from healthy donors [12].

In the crosstalk between cells of the immune system, co-stimulatory molecules play a vital role. Several receptors actively engage to provide stimulation of nearby cells, leading to growth, differentiation and cytokine production. Some of these molecules are also directly involved in the development of autoimmune diseases, since their aberrant expression can support a response directed against self-determinants. CD134, also known as OX40, has been known for years as a co-stimulatory molecule expressed on recently activated T cells. Its role as a critical co-stimulatory molecule is well described [15] and more recently, CD134 was described as directly involved in the reversal of Treg suppression, a phenomenon often observed in autoimmune diseases [16]–[18].

CD154 is crucial to the effector function of CD4 T-cells that co-stimulate CD8 T-cells, macrophages, dendritic cells and B-cells [19] and is regulated by IL-2 and IL-15 on CD4 T-cells [20]. The possible role of CD134 and CD154 in autoimmunity is becoming evident, as CD134 might be involved in RA [21] and CD154 is now emerging as a risk factor in Type 1 Diabetes and RA [22], [23].

In bystander activation, cytokines mediate the stimulation of cells not related to the initial antigen-specific response. Although IL-17A has been described for years to be the prime pro-inflammatory cytokine secreted by CD4 T-cells, others are now emerging, illustrating their diverse and overlapping effects. GM-CSF and IL-22 are both cytokines with a range of effects on Th17 development and function. Both cytokines are induced in Th17-cells by IL-23, produced by activated dendritic cells. GM-CSF in particular has been shown to be critical to the inflammatory potential of Th17 cells, in that IL-23 drives the production of GM-CSF, which in turn stimulates IL-23 production from antigen presenting cells [24], [25]. In particular, Codarri et al. showed that GM-CSF might be even more pro-inflammatory than IL-17A, and they suggest that GM-CSF marks the effector stage of Th17 cells [25]. IL-22 can be directly induced by IL-6 or IL-23 and contributes to inflammation though STAT3 activation, which in keratinocytes is a critical step in the development of psoriasis [26]. IL-22 is found elevated in several autoimmune diseases [27] and it was recently shown to directly exacerbate disease in a rheumatoid arthritis model [28]. GM-CSF is up-regulated in several autoimmune diseases, and is directly responsible for the encephalitogenicity of the Th17 cells mediating disease in an EAE mouse model [24], [25].

In this study, we show that TL1A induces and sustains expression of CD25, CD134, CD154 and LFA-1 on effector CD4^+^ T-cells. We also demonstrate that TL1A specifically induces IL-22 and GM-CSF production from previously activated T-cells. We have previously shown that TL1A induces IL-6 in healthy human leukocytes, but the stimulated effector T-cells are distinct, as no IL-6 is produced.

## Materials and Methods

### Purification and stimulation of lymphocytes

Buffy coats from healthy blood donors were obtained from the Blood Bank at the Copenhagen University Hospital (Denmark), in agreement with the local ethics committee (Region Hovedstaden). PBMC were purified by density centrifugation and incubated for 1 hour with washed pan-mouse beads (Invitrogen, Cat# 11041), and phagocytic cells removed by magnet. Purified PBL were stimulated with CD3/CD28 beads (Invitrogen, Cat # 111.32D) as described by the manufacturer, and cells supplied with media + IL-2 (20 U/mL) as needed.

After 12 days, CD3/CD28 beads were removed by magnet, and the cells set up in new media with 10^6^ cells/mL. For CD8 depletion, Dynabeads Pan Mouse IgG beads (Invitrogen, Cat# 11041) and the CD8α antibody (Cat# 16-0086-81, eBioscience) were used according to manufacturer's protocol. Cytokines, blocking TL1A antibody (TL1AAb) and Cyclosporine A (CsA) were added in the following concentrations: IL-12 (RnD Systems, Cat# 219-IL): 4 ng/mL, IL-15 (Peprotech, Cat# 200-15): 10 ng/mL, IL-18 (MBL, Cat# B003-5): 40 ng/mL, TL1A (RnD Systems, Cat# 1319-TL): 100 ng/mL, TL1AAb (RnD Systems, Cat# MAB7441): 1 µg/mL, CsA (Sigma-Aldrich, Cat# C1832):1 µg/mL.

### Flowcytometry

Cells were briefly washed in cold PBS+5% FBS, and stained for extracellular markers: CD4-APC (Biolegend, Cat# 300514), CD25-PE (302606, Biolegend), CD134-PE (555838, BD Pharmingen), CD154-PE (555700, BD Pharmingen), LFA-1-PE (1433, Immunotech). Cells were analyzed using the BD Accuri C6 flow cytometer and data analyzed using FCS Express vs 3.0.

### Cytokine measurement

Cytokines were measured on supernatants collected ten days after cytokine stimulation. ELISA was performed according to manufacturers' protocol using Human GM-CSF ELISA Ready-SET-Go! (eBioscience, 88–8337) or Human IL-22 ELISA Ready-SET-Go! (eBioscience, 88–7522). IL-6 was measured using the Diaclone Diaplex kit (Nordic Biosite, 880 030 001) according to manufacturer's protocol, using the BD Accuri C6 flow cytometer and data analysed using Flowcytomix Pro (eBioscience).

## Results

### Effect of TL1A on lymphocyte activation

**Figure 1:**
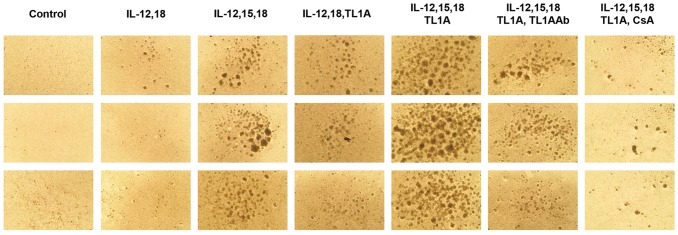
PBLs from three different donors were purified and stimulated using CD3/CD28 beads. After 12 days, beads were removed and the cells set up in new media. Cytokines were added in the following concentrations IL-12: 4 ng/mL, IL-15: 10 ng/mL, IL-18: 40 ng/mL, TL1A: 100 ng/mL, TL1A Ab: 1 µg/mL, CsA 1 µg/mL. Pictures using the light microscope were taken at 25× magnification.

Here we investigated the effect of TL1A on previously activated T cells. Because TL1A is up-regulated in locally inflamed tissues in RA and psoriasis, we suspected that it might have a direct role on the controlling CD4 effector T-cells. We stimulated purified lymphocytes from healthy donors with CD3/CD28 beads and maintained them with low IL-2 levels (20 U/mL) for 12 days. At day 12, these effector T-cells (>99% were CD3^+^ cells, data not shown) were stimulated with different cytokine combinations of IL-12, IL-15, IL-18 and TL1A, and cells observed under light microscope after seven days. Pictures from three different donors, taken seven days after cytokine re-stimulation are shown in Figure 1.

The results clearly show that TL1A is a powerful co-stimulatory molecule for effector T-cells. Large aggregates are visible after TL1A co-stimulation clearly indicating activation and proliferation. This prompted us to investigate the expression of co-stimulatory molecules and secreted cytokines from the activated effector T-cells.

### TL1A induces co-stimulatory molecules on CD4^+^ T cells

It was evident from initial observations that TL1A has a profound effect on previously activated T cells. Cytokine activation of CD4 T cells has not been extensively described, and so we repeated the experiment, focusing our attention on co-stimulatory molecules and cytokine receptors, to measure activation and enhanced responsiveness. We measured expression of several surface molecules after 72 hours.

**Figure 2:**
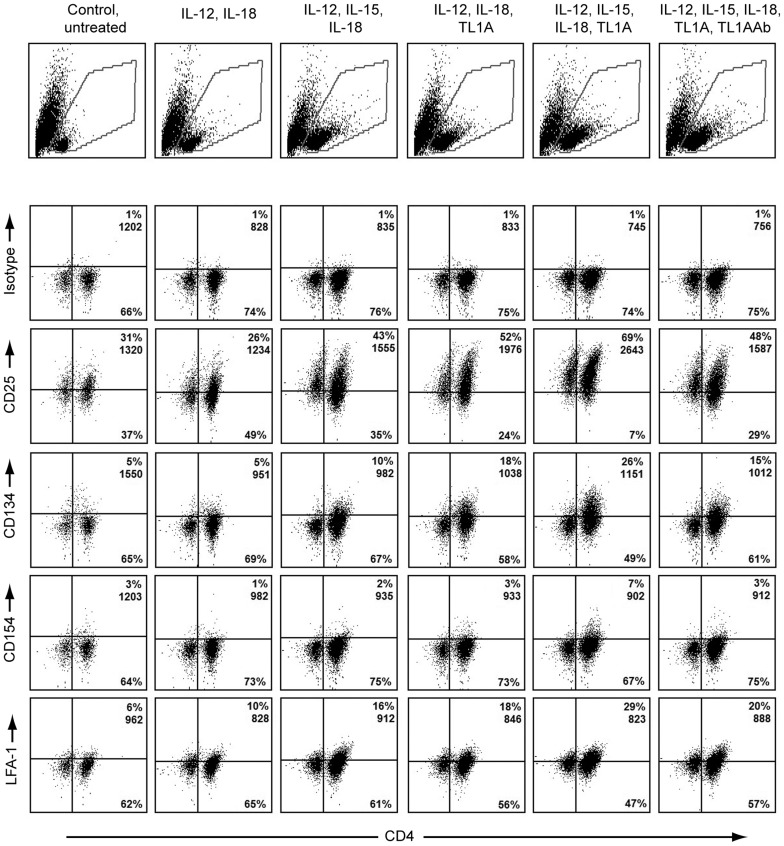
Surface expression of CD4 and different receptors at different time points. Purified PBLs were stimulated for 12 days with CD3/CD28 beads. After 12 days, beads were removed and the cells set up in new media. Cytokines were added in the following concentrations IL-12: 4 ng/mL, IL-15: 10 ng/mL, IL-18: 40 ng/mL, TL1A: 100 ng/mL, TL1A Ab: 1 µg/mL. Surface expression of co-receptors and activation markers CD25, CD134, CD154 and LFA-1 after 72 h. Upper panels: Gating strategy for lymphocytes. Lower panels: Co-staining for CD4 and CD134, CD154, CD25 of LFA-1, % positive cells and MFI for upper right quadrant are shown. Data are representative of results obtained with cells from three different donors.

As shown in Figure 2, the addition of TL1A to the stimulation with IL-12, IL-15 and IL-18 increased the expression of the different surface molecules on CD4 T-cells: CD25 from 52% to 69%, CD134 from 18% to 26%, CD154 from 3% to 7% and LFA-1 from 18% to 29%. Note that although CD154 in this particular donor was not heavily induced, the specific up-regulation was observed in three separate donors.

**Figure 3:**
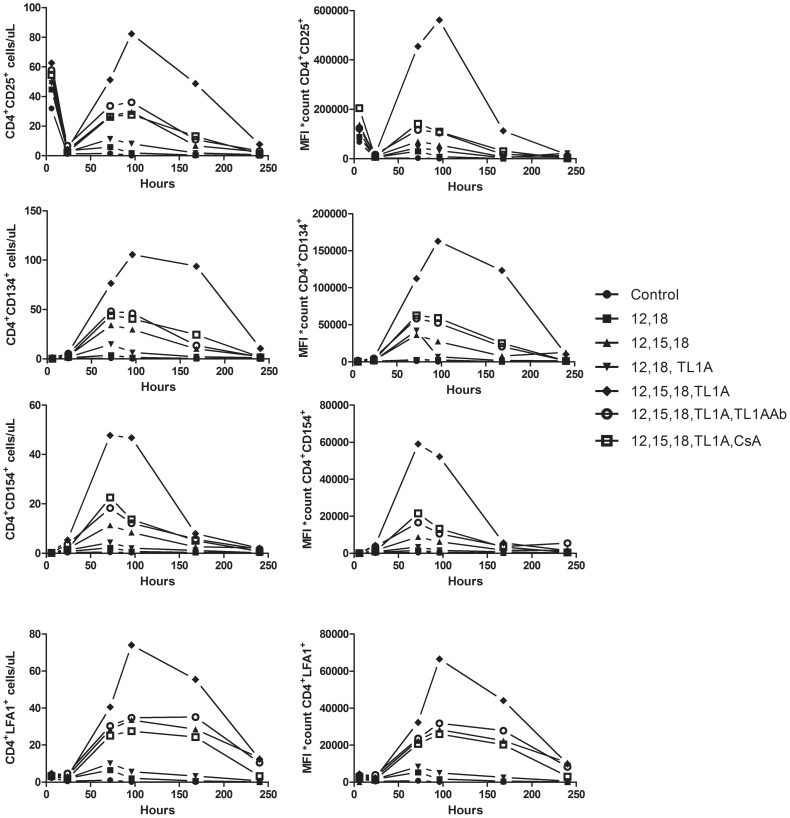
The effect of TL1A on CD4^+^ T cell activation. Purified PBLs were stimulated for 12 days with CD3/CD28 beads. After 12 days, beads were removed and the cells set up in new media. Cytokines/inhibitors were added in the following concentrations IL-12: 4 ng/mL, IL-15: 10 ng/mL, IL-18: 40 ng/mL, TL1A: 100 ng/mL, TL1A Ab: 1 µg/mL, CsA 1 µg/mL. Surface markers were measured at t = 6, 24, 72, 96, 168 and 240 h. Cells/µL was multiplied by MFI (cells/µL*MFI) to take both expression levels and cell growth into account. Data are representative of results obtained with cells from three different donors.

To corporate these results, we examined how TL1A affects surface expression of these co-stimulatory molecules over time. Cyclosporine A (CsA) was included to see if the up-regulation of co-stimulatory molecules was a result of a calcineurin mediated signal. We followed the cells by flow cytometry for a total of 10 days, accumulated data are shown in Figure 3.

Cytokine stimulation resulted in strong up-regulation of CD25 and CD154, peaking after 72–96 h. Interestingly though, both CD134 and LFA-1 were highly expressed even seven days (168 h) after cytokine stimulation, indicating a somewhat delayed and prolonged response. The effect of adding TL1A along with the combination of IL-12, IL-15 and IL-18 was remarkable, and the effect almost completely abolished by addition of a TL1A blocking antibody.

It was evident that TL1A strongly supports the up-regulation of CD25, CD134, CD154 and LFA-1. However, it also became clear that the underlying mechanisms controlling the different surface markers are not the same. Whereas CD25, CD134 and CD154 expression was largely down-regulated by the addition of CsA, LFA-1 expression remained high.

**Figure 4:**
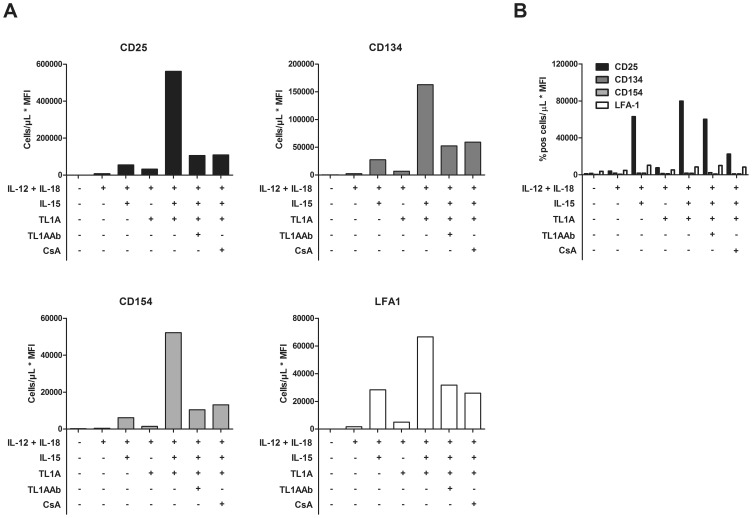
Expression of surface markers on CD4^+^ T-cells after stimulation. A. Purified PBLs were stimulated for 12 days with CD3/CD28 beads, after which cells were stimulated with combinations of cytokines. Cytokines/inhibitors were added in the following concentrations: IL-12: 4 ng/mL, IL-15: 10 ng/mL, IL-18: 40 ng/mL, TL1A: 100 ng/mL, TL1A Ab: 1 µg/mL, CsA: 1 µg/mL Expression of surface markers was measured by flow cytometry after 96 h. Cells/µL was multiplied by MFI (cells/µL*MFI) to take both expression levels and cell growth into account. B. Freshly purified PBLs were stimulated with Cytokines/inhibitors in the following concentrations: IL-12: 4 ng/mL, IL-15: 10 ng/mL, IL-18: 40 ng/mL, TL1A: 100 ng/mL, TL1A Ab: 1 µg/mL, CsA: 1 µg/mL. Expression of surface markers was measured by flow cytometry after 72 h. Please note that the Y-axis of the graphs differ in range. Data are representative of results obtained with cells from three different donors.

We also compared the effect of TL1A on extracellular markers in freshly purified leukocytes with that in effector T-cells. The results are shown in Figure 4 and clearly depict the difference between freshly purified and previously stimulated CD4 T-cells. Whereas effector CD4 T-cells up-regulate several extracellular markers in response to cytokine activation (Figure 4A), the CD4 T-cells in freshly purified leukocytes only respond with CD25 up-regulation, low levels of LFA-1 and no up-regulation of CD134 or CD154 (see Figure 4B).

### TL1A induces GM-CSF and IL-22 from primed CD4 T-cells

We have previously shown that TL1A induces IL-6 production from freshly purified leukocytes [12].To examine if a similar phenotype was present in effector T-cells, twelve-day old TCR-activated T-cells were re-stimulated with cytokine combinations, and different pro-inflammatory cytokines measured after 96 h.

**Figure 5:**
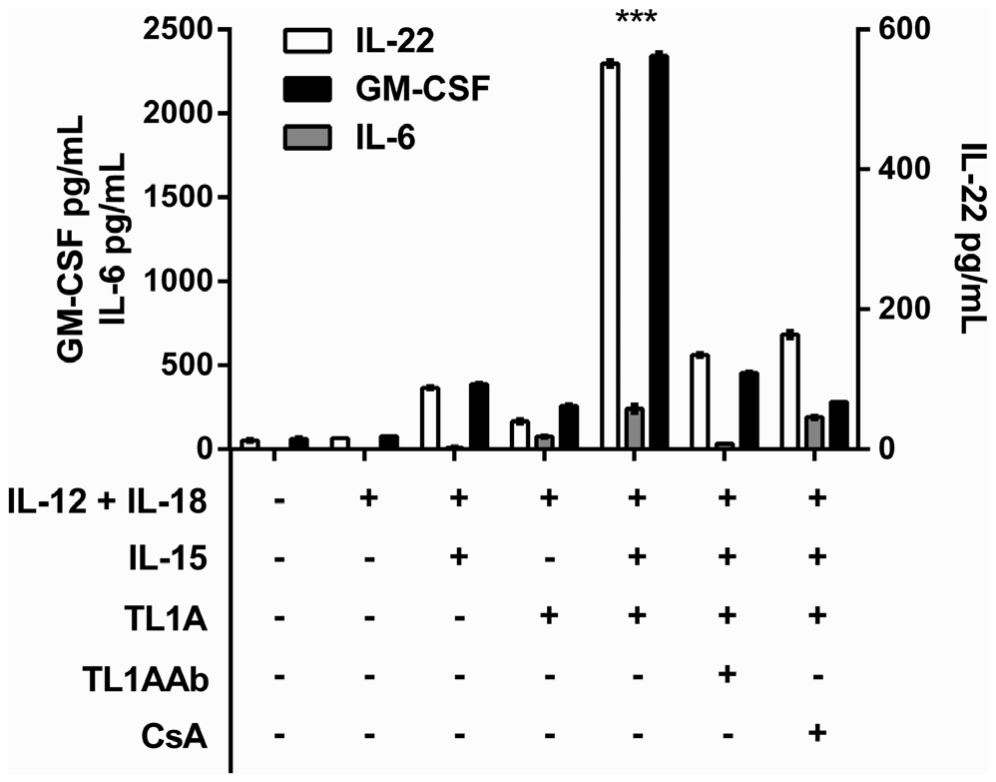
TL1A specifically induces IL-22 and GM-CSF but no IL-6 in primed T cells. Purified PBLs were stimulated for 12 days with CD3/CD28 beads. After 12 days, beads were removed and the cells set up in new media. Cytokines/inhibitors were added in the following concentrations IL-12: 4 ng/mL, IL-15: 10 ng/mL, IL-18: 40 ng/mL, TL1A: 100 ng/mL, TL1A Ab: 1 µg/mL, CsA 1 µg/mL Supernatant was collected 96 h after stimulation and cytokines measured by ELISA or multiplex as described in Materials and Methods. Mean +/− SEM on two measurements is shown. The addition of TL1A significantly increased the expression of cytokines compared to all other cytokine combinations tested;α IL-22 (P<0.0002) and GM-CSF (P<0.00005), t-test. Data are representative of results obtained with cells from three different donors.

**Figure 6:**
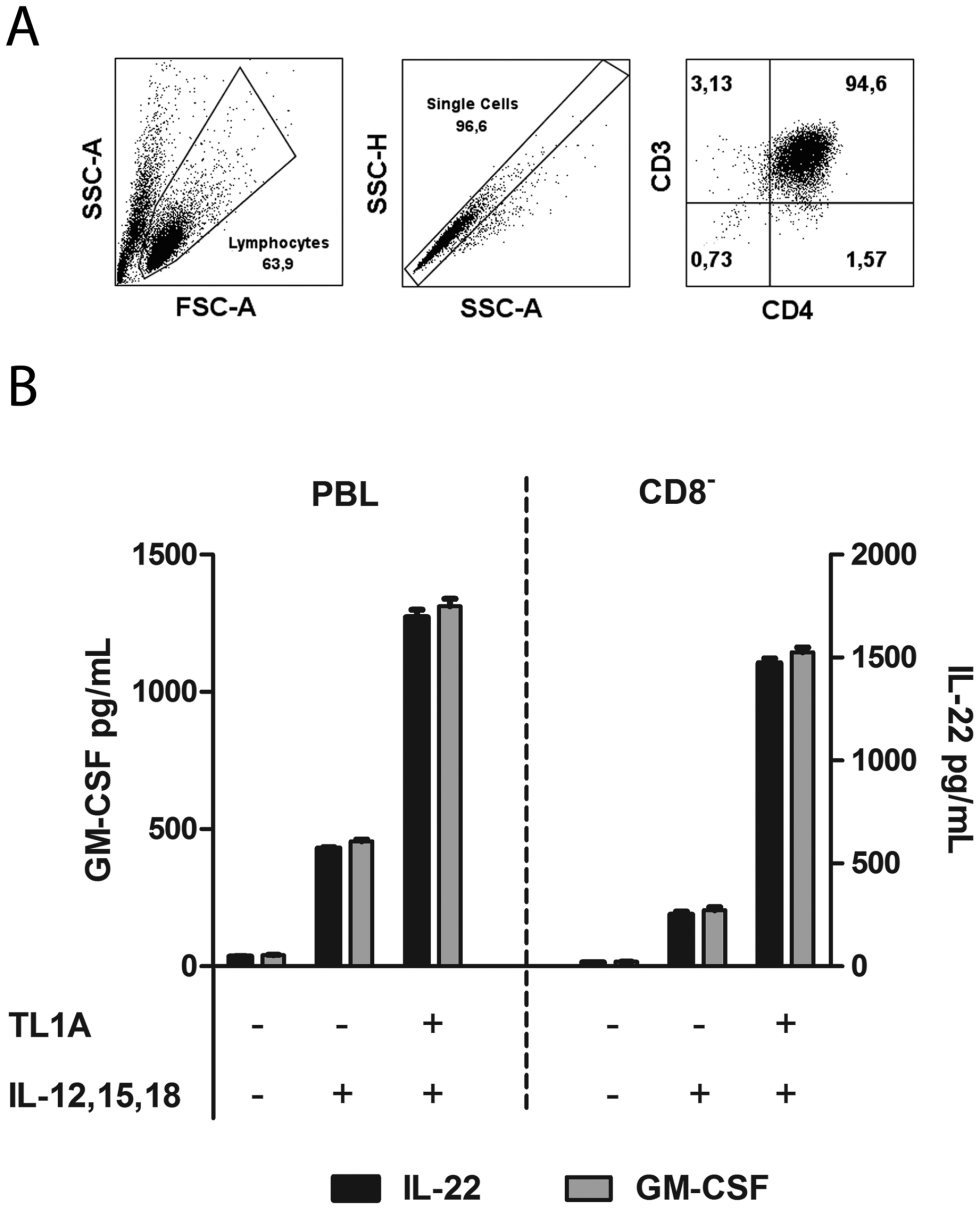
IL-22 and GM-CSF is produced by CD8^−^ T-cells. Purified PBLs were stimulated for 12 days with CD3/CD28 beads. After 12 days, beads were removed and the cells set up as PBLs or CD8 depleted PBLs in new media. A. Left panel: Gating strategy for lymphocytes. Middle panel: Single cells. Right panel: Verification of CD8 depletion by staining for CD4/CD3 (∼95% were CD3^+^CD4^+^). B. IL-22 and GM-CSF production by PBLs and CD8 depleted cells from A. Supernatant was collected seven days after stimulation and cytokines measured by ELISA as described in Materials and Methods. Cytokines/inhibitors were added in the following concentrations IL-12: 4 ng/mL, IL-15: 10 ng/mL, IL-18: 40 ng/mL, TL1A: 100 ng/mL. Mean +/− SEM on two measurements is shown. Data are representative of results obtained with cells from two different donors.

As shown in Figure 5, a strong and TL1A dependent induction of IL-22 and GM-CSF was observed, but there was little or no IL-6 production. We also measured IL-7 and IL-17, but they were undetectable in three different donors (data not shown). To verify that IL-22 and GM-CSF were in fact induced in CD4 effector T-cells, we depleted stimulated PBLs for CD8 T-cells (leaving ∼95% CD4 T-cells) and stimulated these with different cytokine combinations. IL-22 and GM-CSF production remained largely unaffected, showing that both cytokines can be produced by CD4 effector T-cells (Figure 6).

To summarize, TL1A does not induce IL-6 production in effector T-cells like we previously observed in freshly purified leukocytes. Most interestingly, even though no IL-6 was present, high levels of IL-22 and GM-CSF were produced by CD4 effector T-cells, together with a prolonged up-regulation of CD134, CD154, CD25 and LFA-1. This indicates that at least two different populations are stimulated by TL1A: One is TCR-independent, is present in freshly purified leukocytes and produces IL-6 in response to stimulation. The other population, which includes CD4 effector T-cells, is present 12 days after TCR stimulation, and produces GM-CSF and IL-22 in response to TL1A stimulation. The effect of TL1A on CD134, CD154 and CD25 expression as well as IL-22/GM-CSF production was specific and could largely be abrogated by addition of CsA.

## Discussion

In an autoimmune setting, many factors contribute to the maintenance of inflammation. Cytokines and chemokines made by antigen-presenting cells can attract and activate lymphocytes, which in turn provide more co-stimulatory signals, both by cytokine production and cell-cell contact. Breaking down the cytokine networks that keep inflammation going is one of the major challenges in treatment of chronic inflammation. Most likely, several factors need to be blocked to completely abolish an ongoing inflammation, but if we can specify these factors, we might be able to provide more effective treatment with less side effects. Since TL1A is not detected under normal conditions, only in inflammatory settings, it might just be one of these factors.

Here we show that TL1A together with IL-12, IL-15 and IL-18 strongly enhances and prolongs expression of CD25, CD134, CD154 and LFA-1 on effector CD4^+^ T-cells. We further show that this cytokine combination, specifically dependent on TL1A, induces production of GM-CSF and IL-22 from effector CD4 T-cells. Our findings thus implicate a role for TL1A both on innate responses as well as on adaptive responses. GM-CSF and IL-22 are both emerging as key players in autoimmunity, sometimes more potent than IL-17A in the development of disease [24], [28]. Hence, the induction of these two cytokines alone could justify considering TL1A as a target for blocking in autoimmune diseases. On top of this, TL1A induces and prolongs several co-stimulatory surface markers on CD4^+^T-cells. The increased expression of CD134 and CD154 likely results in easier interaction with monocytes, macrophages, dendritic cells and B-cells, and the increased expression of CD25 and LFA-1 increases IL-2/IL-15 sensitivity and cell-cell contact. Although the expression of CD25 could be a result of Treg proliferation, their suppressive function would probably be attenuated by the high levels of IL-6 present in inflammation [29].

IL-15 and IL-2 are both cytokines that stimulate growth, but their targets and origin differ. IL-2 is produced by T-cells in direct response to TCR stimulation, whereas IL-15 is produced by APCs and different tissue-specific cell types. One of our initial findings was that re-stimulation of effector T-cells using TL1A was only possible if the level of IL-2 during growth was kept low (20 U/mL instead of 100 U/mL normally used), corresponding to the observation made by Bamias et al. in 2006, that IFN-γ release mediated by TL1A was higher in cells stimulated with low levels of anti-CD3 [30]. This setting could reflect conditions in chronic inflammation tissue such as joints in RA or psoriatic lesions. We believe that this particular cytokine activation is mainly possible when T-cells receive inadequate or weak stimulation, such as through IL-15. The strong stimulation received by high levels of IL-2 probably results in Th1 differentiation, eliminating effect of weaker co-stimulatory signals.

The emerging understanding of cytokine networks is currently transforming treatment strategies for autoimmune diseases such as RA and Psoriasis. However, these cytokine networks also spawn a series of questions about the reactions preceding pro-inflammatory cytokines. Our current study supports the hypothesis previously presented, that TL1A takes part in initiation and maintenance of inflammation [8], [12], [14], [31].

## Supporting Information

Rawdata S1 **Flowcytometry .c6 rawdata underlying** **Figure 2** **.**
(ZIP)

Rawdata S2 **Flow cytometry rawdata .c6 file for figure 3 containing surface expression measured after 6 hours.**
(ZIP)

Rawdata S3 **Flow cytometry rawdata .c6 file for figure 3 containing surface expression measured after 24 hours.**
(ZIP)

Rawdata S4 **Flow cytometry rawdata .c6 file for figure 3 containing surface expression measured after 72 hours.**
(ZIP)

Rawdata S5 **Flow cytometry rawdata .c6 file for figure 3 containing surface expression measured after 96 hours.**
(ZIP)

Rawdata S6 **Flow cytometry rawdata .c6 file for figure 3 containing surface expression measured after 168 hours.**
(ZIP)

Rawdata S7 **Flow cytometry rawdata .c6 file for figure 3 containing surface expression measured after 240 hours.**
(ZIP)

Rawdata S8 **Rawdata from S1–S6 exported to GraphPad prism and presented in graphs, including** **Figure 3** **.**
(PZF)

Rawdata S9 **Rawdata from S4 exported to prism, underlying** **Figure 4A** **.**
(PZF)

Rawdata S10 **Flowcytometry .c6 rawdata underlying** **figure 4B** **.**
(ZIP)

Rawdata S11 **Rawdata from S9 exported to GraphPad prism and presented in graphs, including** **figure 4B** **.**
(PZF)

Rawdata S12 **Rawdata in GraphPad prism presented in graphs, including** **figure 5** **.**
(PZF)

Rawdata S13 **Flowcytometry .c6 rawdata from depletion studies as shown in** **Figure 6A** **.**
(ZIP)

Rawdata S14 **GraphPad prism rawdata for** **Figure 6B** **.**
(PZF)
